# Supplementary material for: The fossil Osmundales (Royal Ferns)—a phylogenetic network analysis, revised taxonomy, and evolutionary classification of anatomically preserved trunks and rhizomes
Source: PeerJ. 2017 Jul 11;5:e3433. doi: 10.7717/peerj.3433 (PMC5508817; doi:10.7717/peerj.3433)
Supplement: Supplemental Information 1 [file peerj-05-3433-s001.pdf]

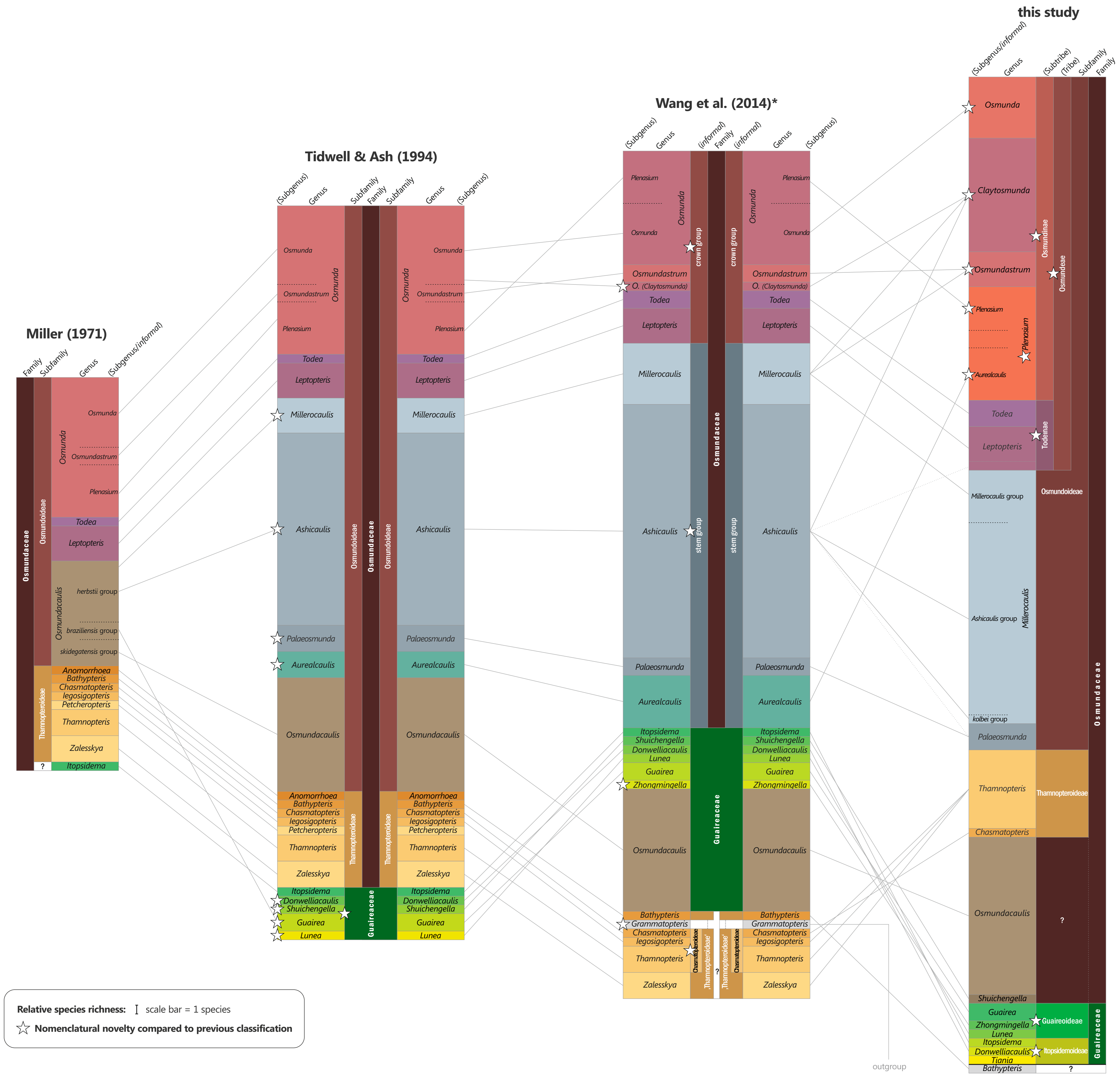

Figure S1. Nomenclatural and taxonomic history and taxic diversity of extinct and extant Osmundales, based on selected comprehensive reviews since the 1970s.

*This analysis is based partly on „genus-level composites" as operational units whose coding was derived either directly from one particular, presumably representative species or via composite coding of information from a varying range of selected species. We inferred the number of species within each of these composites (indicated here by the height of the relevant box) by summarizing the number of valid species known at the time of publication for the particular composite as circumscribed by the authors.
