# Supplementary figures and images for: The fossil Osmundales (Royal Ferns)—a phylogenetic network analysis, revised taxonomy, and evolutionary classification of anatomically preserved trunks and rhizomes
Source: PeerJ. 2017 Jul 11;5:e3433. doi: 10.7717/peerj.3433 (PMC5508817; doi:10.7717/peerj.3433)

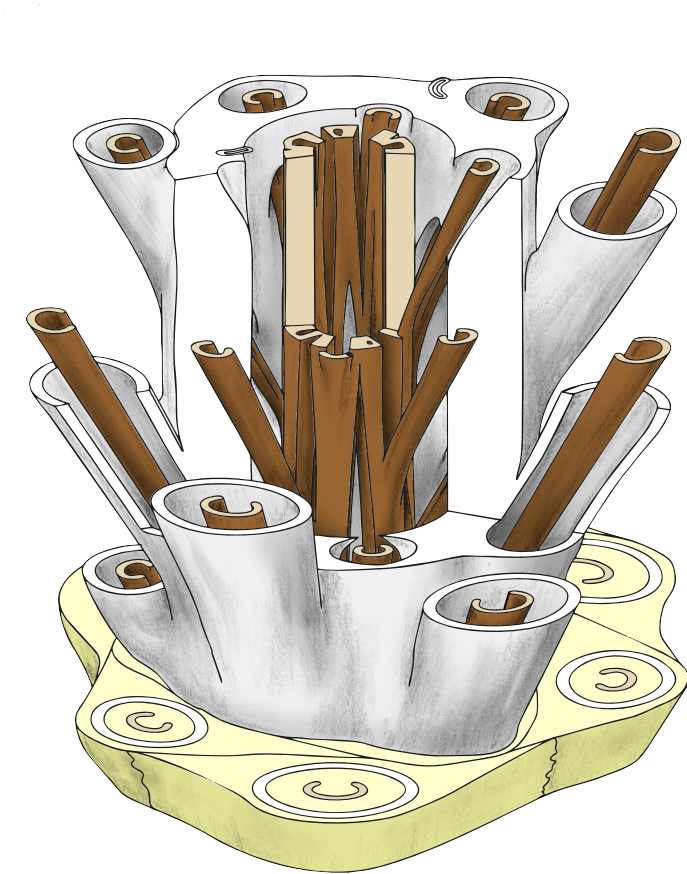

Supplement: Supplemental Information 2 — Petiolar parenchyma in yellow-green; outer and inner surface of sclerenchymatic outer cortex in light grey; xylem in brown; parenchyma of pith and inner cortices left transparent and most roots omitted for clarity reasons (unlabelled version of Fig. 1). [file peerj-05-3433-s002.pdf]

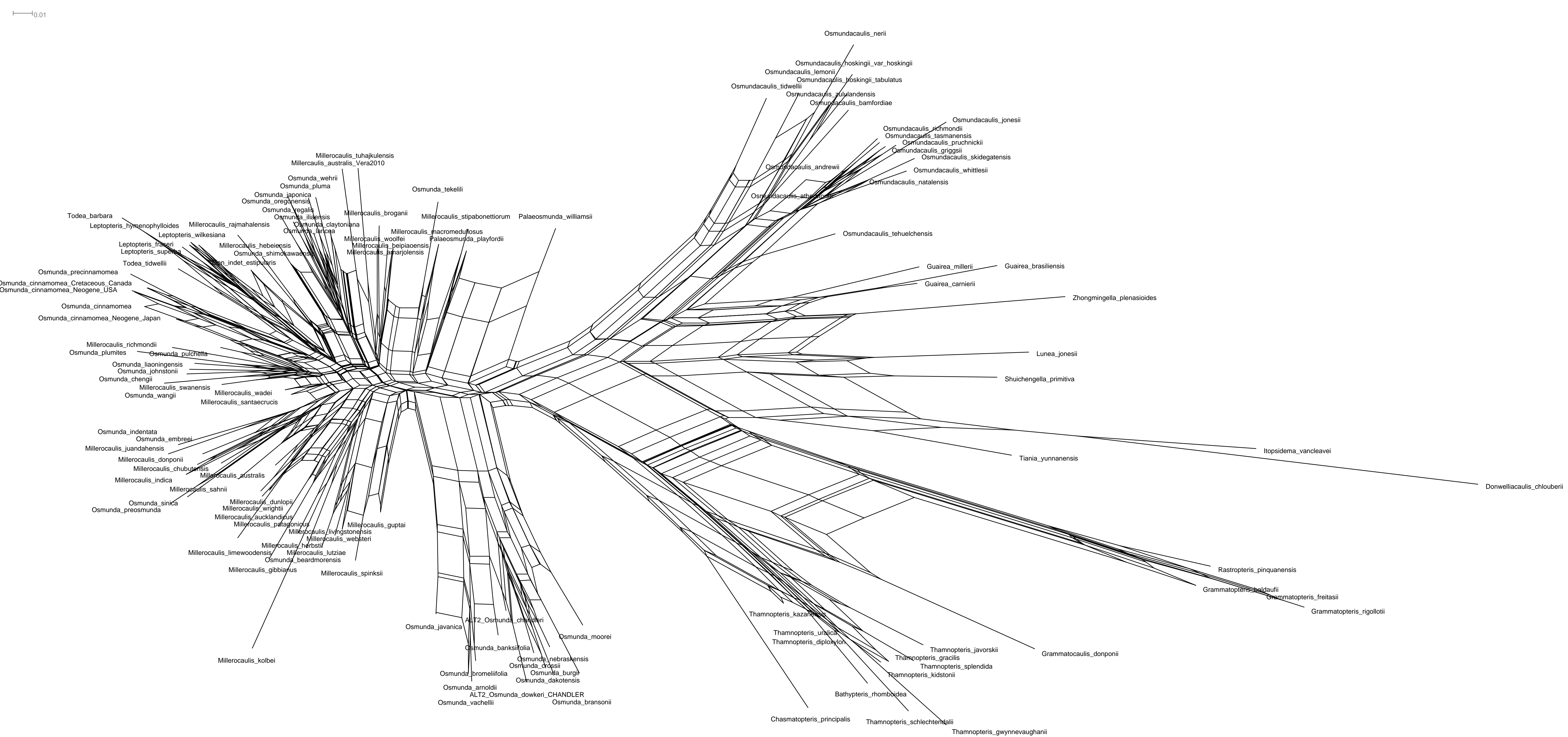

Supplement: Supplemental Information 3 — Fully labelled version of Fig. 9. [file peerj-05-3433-s003.pdf]

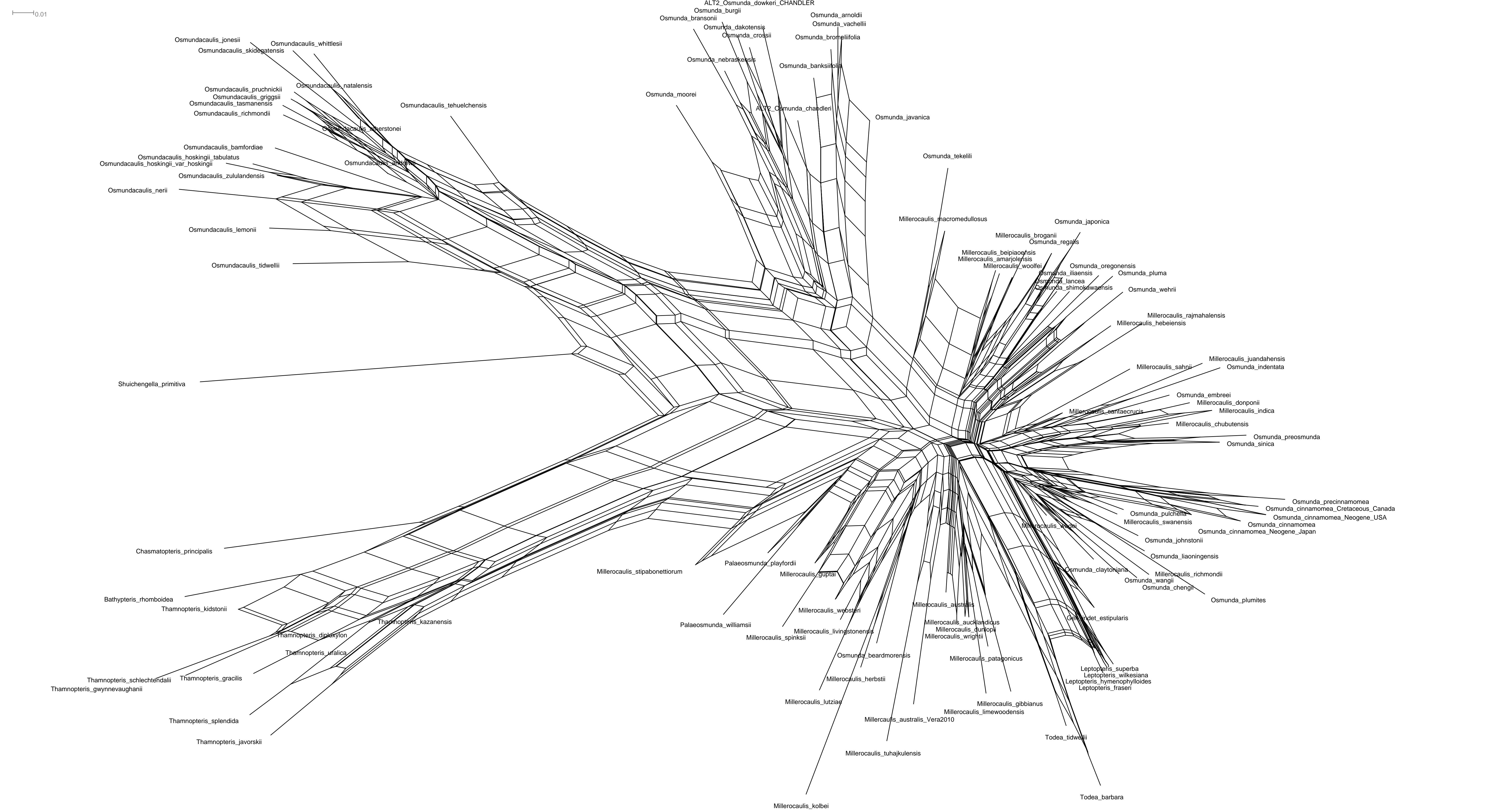

Supplement: Supplemental Information 4 — Fully labelled raw version of Fig. 11. [file peerj-05-3433-s004.pdf]

0.01

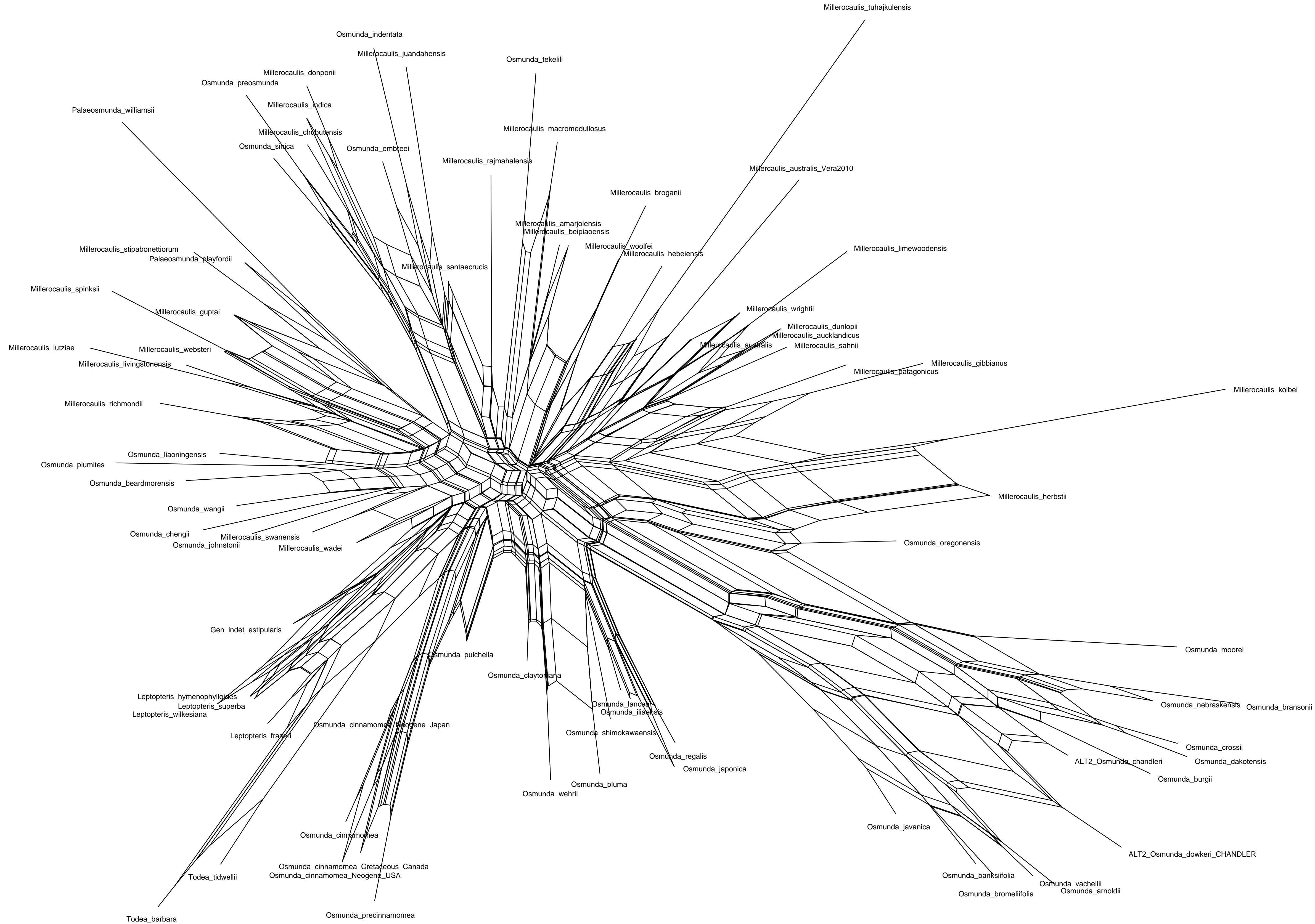

Supplement: Supplemental Information 5 — Fully labelled raw version of Fig. 13. [file peerj-05-3433-s005.pdf]

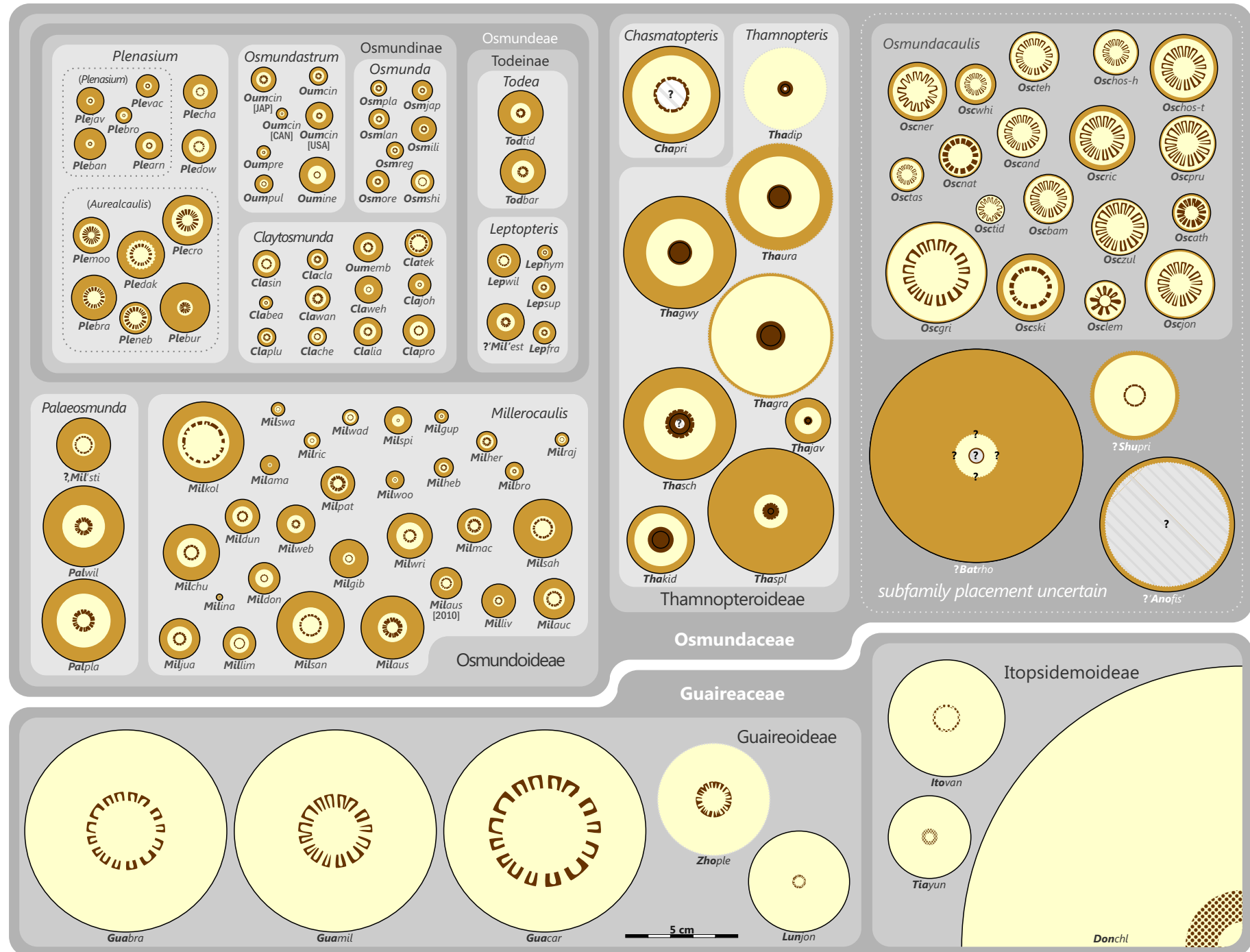

Supplement: Supplemental Information 6 — Each circular diagram represents a simplified stem cross-section of the relevant species that gives basic information on the structure and proportional sizes of the stem core, xylem siphon, cortex or cortical layers and the entire stem excluding the mantle, all to the same scale. Taxon names are given in the form of six-letter labels that are contractions formed from the three first letters of the genus name in bold followed by the first three letters of the specific epithet; Oum, Osmundastrum. Primarily parenchymatic tissues in pale yellow; stelar metaxylem in dark brown; sclerenchyma in light brown; note that individual fibres or fibre patches in pith or cortical tissues are omitted. [file peerj-05-3433-s006.pdf]
