# Supplementary material for: The fossil Osmundales (Royal Ferns)—a phylogenetic network analysis, revised taxonomy, and evolutionary classification of anatomically preserved trunks and rhizomes
Source: PeerJ. 2017 Jul 11;5:e3433. doi: 10.7717/peerj.3433 (PMC5508817; doi:10.7717/peerj.3433)
Supplement: Supplemental Information 11 [file peerj-05-3433-s011.pdf]

Tutorial and example assessment: Classification and first phylogenetic placement of new-described Osmundales rhizomes

Step 1: Applying the key.

Using several diagnostic characters, the systematic affinity of the fossil can be established via the multifurcating key. This is illustrated here (Fig. S5-1) for the newly described *Millerocaulis zamunerae* Sagasti, García Massini, Escapa et al. (Sagasti et al., in press). The diagnostic characters of *M. zamunerae* confirm the original placement in the extinct genus *Millerocaulis* as understood also here. Depending on the preservation of the fossil and the characters that can be observed, it may be that the fossil cannot be securely placed in a single taxon. Nevertheless, in most cases, the key should at least allow narrowing down the groups/lineages to which a rhizome fossil can be assigned.

Step 2: Adding the fossil to the reference matrix.

The reference matrix is provided in the Supplemental Materials. We recommend adding any new taxa and, if feasible, new discriminative characters to this matrix, and freely distribute the updated matrix. Taxa and characters can be added using a range of programs. Here, we use MESQUITE (Maddison & Maddison 2011), a platform-independent freeware, with good annotation capabilities. Our NEXUS-formatted reference matrix has been generated for and optimized for this program.

Open the reference matrix in Mesquite. The file includes the morphological matrix called ‘Miller, emended’ and a genetic matrix (Metzgar et al. 2008). Show the character matrix ‘Miller, emended’ (if you take our provided version, this sheet will be already on top); add a new taxon using the ‘Add Taxa’ tool on the left of the matrix window. Activate the ‘Edit’ tool. Now you can edit the cells of the new taxon. Clicking in a cell will show you the states available for the corresponding character in the shaded area below the matrix. Score all characters that can be observed in the fossil; you can add additional information in the white remarks area below the matrix. Polymorphic characters, a taxon showing one state and another, can be entered as e.g. “0&1” and will exported as “{0 1}” when the NEXUS format is selected; ambiguous characters, where it is unclear whether it is, e.g. state 0 or 1 as “0/1”and will be exported as “(0 1)”. Note that for standard analysis, including those used here, there is no difference between “0&1” and “0/1”.

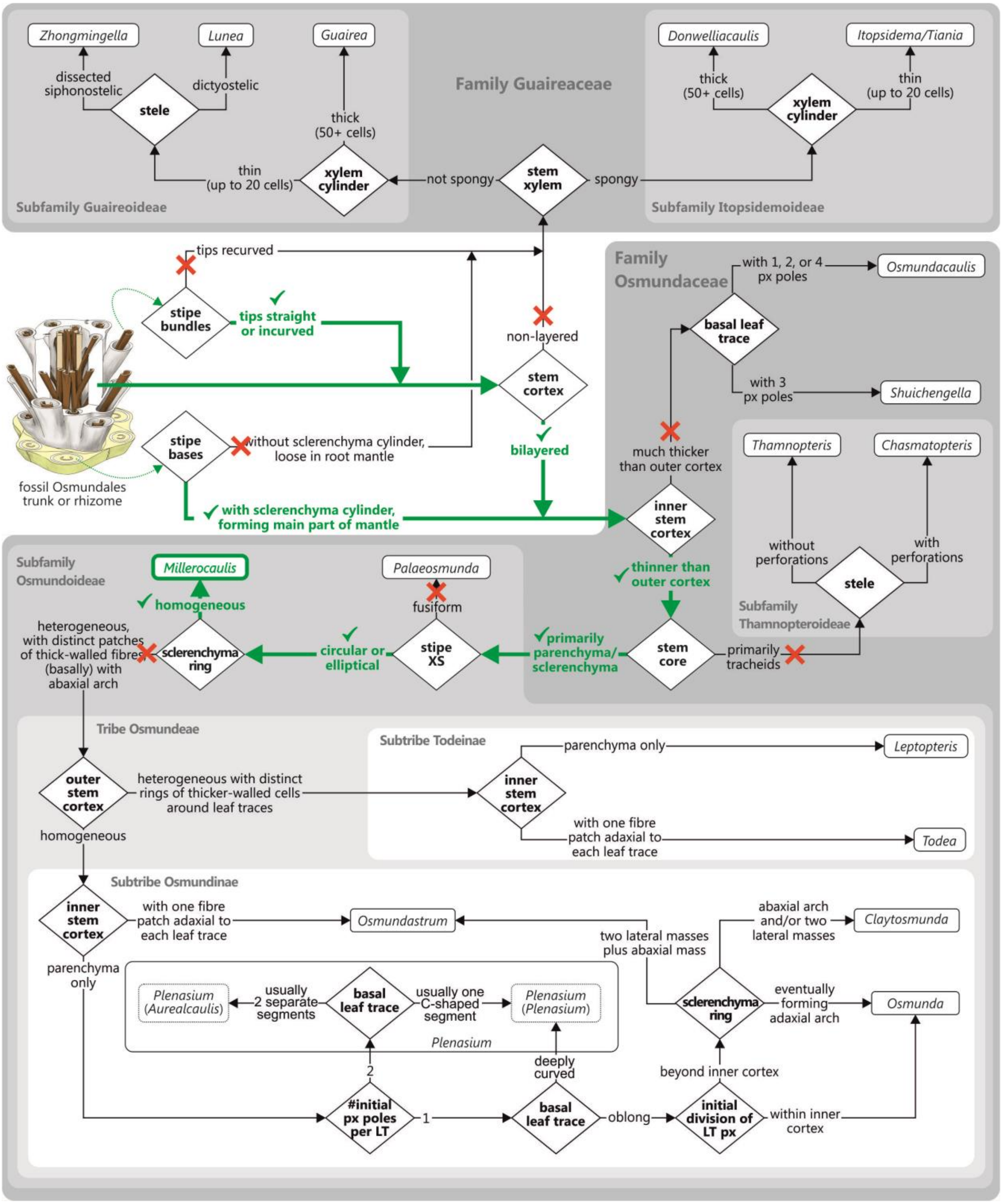

Fig. S5-1. Application of the multifurcating key using the recently described *Millerocaulis zamunerae* as example.

For one-to-one comparison with putative close(st) relatives, move the new taxon to its time-scale and group/lineage as informed by the key. In MESQUITE this can be done by opening the ‘Taxa’ window (e.g. via the T-button; already active in the provided NEXUS file): Select the new taxon to move and move it by typing CTRL-M or drag-and-drop.

Adding new characters. Add new characters using the ‘Add Characters’ tool. Make sure to directly define the states by clicking on the S-icon directly below the matrix window. This opens a new window with a character list and columns defining what “0”, “1”, etc. stand for (already activated in the provided NEXUS file). We recommend avoiding characters with more than three states. If you want to code a complex morphological trait use instead a series of binary (or ternary) characters. Keep in mind that this matrix primarily serves diagnostic, taxonomic-systematic purposes.

Step 3: From character matrix to distance matrix

Using MESQUITE’s export functions, the ‘Miller, emend.’ matrix can be exported as simplified NEXUS format, which can be directly read-in by all NEXUS-compatible programmes. In this example, we use PAUP* (Swofford 2002) to calculate the pairwise distances, and SPLITSTREE (Huson & Bryant 2006) to infer and visualise the resultant neighbour-net splits graphs. The new taxa have been moved to the end, to avoid having re-setting the taxon sets. Exporting a matrix as simplified NEXUS format from Mesquite opens a dialogue window which allows including additional code lines (an example block is provided in the *.simple.nex file in the supplement).

The following code lines need to be added via this window for the here described workflow[1]:

```
BEGIN ASSUMPTIONS;
TaxSet Permian = 1-24;
TaxSet Triassic = 25-33;
TaxSet Jurassic = 34-72 129; [add 131 here to include M. zamunerae]
TaxSet Mesozoic_unclear = 73-76;
TaxSet Cretaceous = 77-97 130;
TaxSet Paleogene = 98-108;
TaxSet Neogene = 109-113;
TaxSet Extant = 114-128;
TaxSet ManyMissing = Anomorrhoea_fischeri Osmundacaulis_janae Osmunda_kidstonii
Osmunda_nathorstii Todea_papuana; [> 60% undefined characters]
TaxSet Grammatopteris_group = 4-6 12 48;
TaxSet Guaireaceae = 7-9 23 24 29 30 49;
TaxSet Thamnopteridoideae = 2 3 14-22; [including Bathypteris]
TaxSet Permian_Osmundoideae = 1 10 11;
TaxSet Osmundacaulis_lineage = 13 62-72 74-76 93-96 129; [including Shuichengella]
TaxSet Millerocaulis_complex = 26-28 32-47 50-54 78-85 130; [add 131 here to include M zamunerae]
TaxSet Osmundeae = 25 31 55-61 73 77 86-92 97-128;
TaxSet Osmunda = 25 31 55-61 73 86-92 98-122;
TaxSet Aurealcaulis = 87-89 98-100 104; [including O. chandleri]
TaxSet Plenasium = 101-103 116-119;
TaxSet Osmundoideae = Permian_Osmundoideae Millerocaulis_complex Osmundeae;
TaxSet Osmundaceae = Thamnopteridoideae Osmundacaulis_lineage Osmundoideae;
END;

BEGIN PAUP;
DEFAULTS SAVEDIST Format=Nexus Triangle=Both Undefined=Asterisk;
DSET Distance=Mean MissDist=Ignore Objective=LSFit;
END;

Delete all;
Restore Osmundoideae Millerocaulis_zamunerae;
Delete ManyMissing ALT1_Osmunda_dowkeri_MILLER;
Exclude Constant;
SaveDist File=OsmoideaePlusMZam.mean.dist;
Delete all;
Restore Millerocaulis_complex Millerocaulis_zamunerae;
Exclude Constant;
SaveDist File=MilcauPlusMZam.mean.dist;
Delete all;
Restore Jurassic Millerocaulis_zamunerae;
Delete ManyMissing ALT1_Osmunda_dowkeri_MILLER;
Exclude Constant;
SaveDist File=JurassicPlusMZam.mean.dist;

[END OF CODE]
```

[1] Note: When new taxa are added and kept within the taxon list and not at the end, the numbers for the taxon sets need to be adapted; if just kept at the end before export from Mesquite, the new number can be added to the existing list (as done here for taxa # 129, 130, not included in our original analysis; and #131, the "new" taxon to be placed in this example). Setting 'SaveDist Undefined' to 'Asterisk' ensures that a matrix will trigger an error message when open in SplitsTree; if this happens remove the taxa from the matrix that have no character overlap with the new taxon/taxa (pairwise distances will not be meaningful in such cases).

Step 4: Inference of planar phylogenetic networks.

The three NEXUS-formatted distance matrices (*.dist) generated in **Step 3** files can be opened with SPLITSTREE or further processed with R using the functions included in the PHANGORN library (Schliep 2011; Schliep et al. 2017). SPLITSTREE will automatically infer a neighbour-net (NNet) splits graph from each distance matrix, when opening the *.dist file. As seen in the Osmundoideae NNet (Fig. S5-2), the new *Millerocaulis zamunerae* groups with other *Millerocaulis* and Osmundeae in the according part of the graph, confirming the identification via the key. According to the *Millerocaulis* NNet (Fig. S5-3), the species is overall close to *Millerocaulis patagonicus* and *Millerocaulis australis*; the rhizome is not an atypical Jurassic representative of this basal, extinct genus.

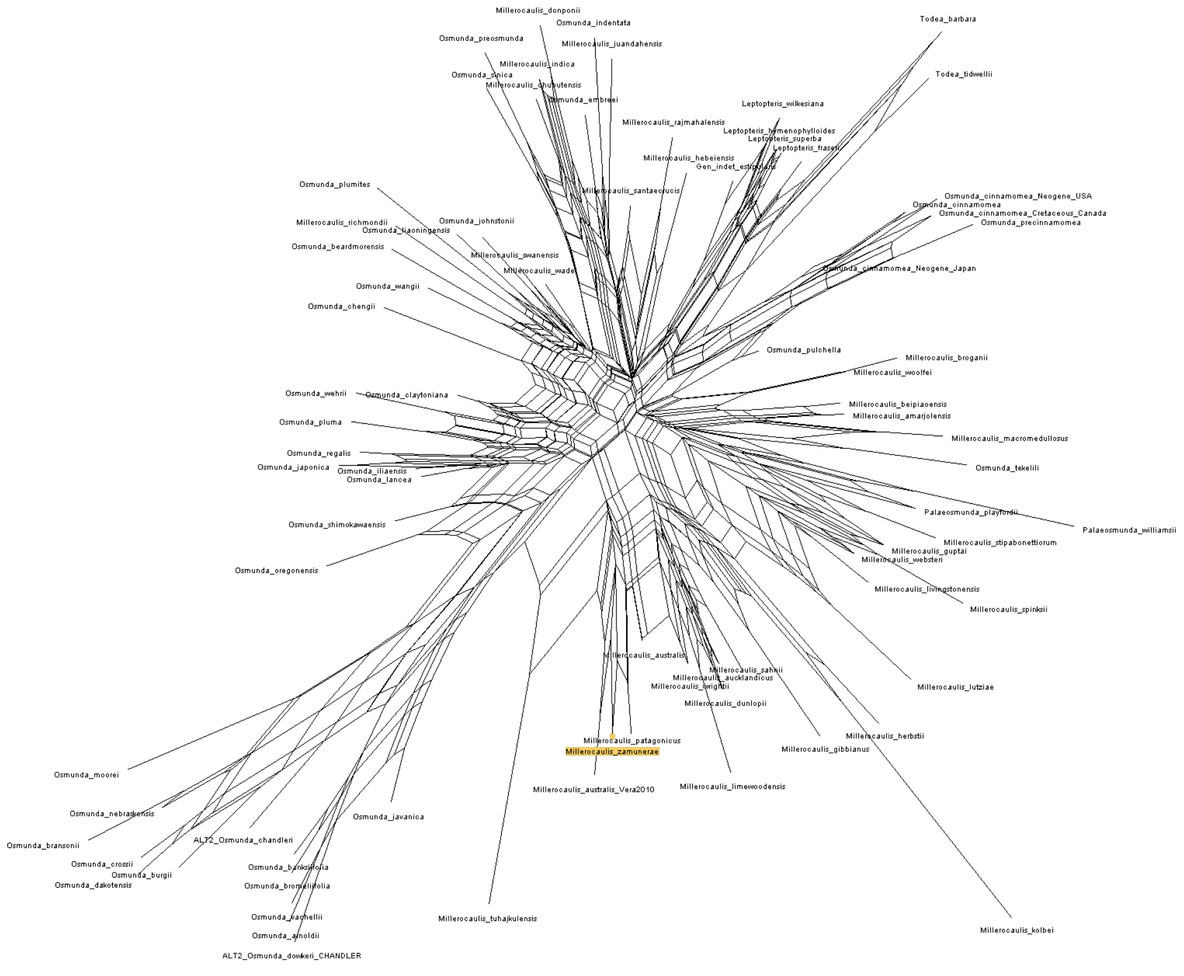

Fig. S5-2. Neigbour-net of all Osmundoideae generated using the above-described workflow (Steps 3 and 4). The position of the newly included *Millerocaulis zamunerae* is highlighted in yellow.

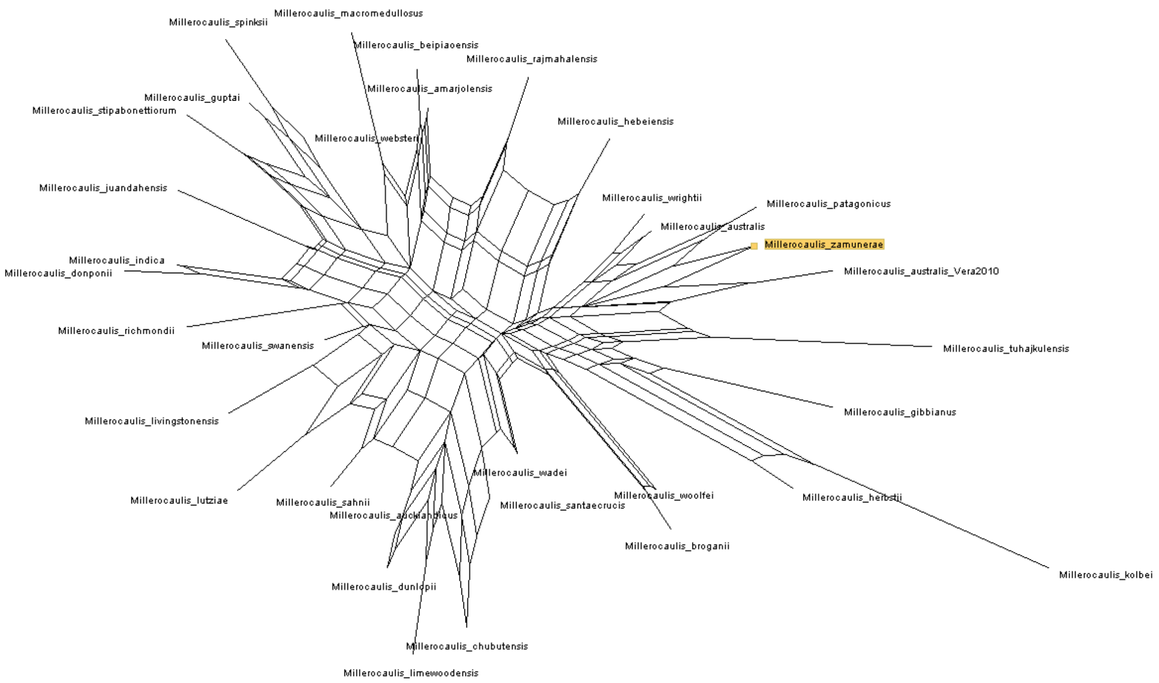

Fig. S5-3. Neigbour-net including all members of genus *Millerocaulis* generated using the above-described workflow (Steps 3 and 4). The position of the newly included *Millerocaulis zamunerae* is highlighted in yellow.

Final step 5: Pairwise comparison with closely related, already described taxa.

Based on the position in the neighbour-net the potentially closest relatives of the new taxon can be identified, and pairwise comparison of the new taxon to its allies at hand of the matrix (and additional observation not scored for the matrix) can aid in putting together the final diagnosis for the new taxon.

References.

Huson DH, Bryant D. 2006. Application of phylogenetic networks in evolutionary studies. *Molecular Biology and Evolution* 23:254-267.

Maddison WP, Maddison DR. 2011. Mesquite: a modular system for evolutionary analysis. 2.75 ed.

Metzgar JS, Skog JE, Zimmer EA, Pryer KM. 2008. The paraphyly of *Osmunda* is confirmed by phylogenetic analyses of seven plastid loci. *Systematic Botany* 33:31–36.

Sagasti AJ, García Massini J, Escapa IH, Guido DM, Channing A. In press. *Millerocaulis zamunerae* sp. nov. (Osmundaceae) from Jurassic, geothermally influenced, wetland environments of Patagonia, Argentina. Alcheringa 40:xxx–xxx.

Schliep K, Potts AJ, Morrison DA, Grimm GW. 2017. Intertwining phylogenetic trees and networks. *Methods in Ecology and Evolution* DOI:10.1111/2041-210X.12760.

Schliep KP. 2011. Phangorn: phylogenetic analysis in R. *Bioinformatics* 27:592–593.

Swofford DL. 2002. PAUP*: Phylogenetic Analysis Using Parsimony (and Other Methods). 4.0 beta 10 ed. Sunderland, MA: Sinauer Associates.
