# Supplementary material for: The fossil Osmundales (Royal Ferns)—a phylogenetic network analysis, revised taxonomy, and evolutionary classification of anatomically preserved trunks and rhizomes
Source: PeerJ. 2017 Jul 11;5:e3433. doi: 10.7717/peerj.3433 (PMC5508817; doi:10.7717/peerj.3433)
Supplement: Supplemental Information 13 [file peerj-05-3433-s013.docx]

**Table S3.** Comparison of treelikeliness of selected morphological matrices as measured in matrix Delta Value (mDV) and individual Delta Values (iDV) ranges.

| Study | Group covered | Matrix dimensions  (taxa x characters) | Fossil taxa included | mDV | iDV range |
| --- | --- | --- | --- | --- | --- |
| Stevenson (1990)^a^ | Cycadales | 12 x 30 | Extant only | 0.21 | 0.15–0.29 |
| Manos et al. (2007)^b^ | Juglandaceae | 33 x 64 | 5 | 0.22 | 0.18–0.31 |
| Jordal et al. (2002) | *Coccotrypes* | 32 x 31 | Extant only | 0.28 | 0.22–0.40 |
| Gandolfo, Nixon & Crepet (2004) | Nympheaceae | 9 x 69 | 1 | 0.30 | 0.24–0.36 |
| Friis et al. (2007) | Spermatophytes | 50 x 102 | 26 | 0.31 | 0.28–0.35 |
| Grimm (1999) | Cycadales | 22 x 40 | 11 | 0.33 | 0.28–0.35 |
| Lehtonen & Myllys (2008) | *Echinodorus* | 70 x 86 | Extant only | 0.33 | 0.26–0.38 |
| Beyra Matos & Lavin (1999) | Aeschynomeneae | 42 x 50 | Extant only | 0.34 | 0.28–0.43 |
| Bortiri, Van den Heuvel & Potter (2006) | *Prunus* | 45 x 25 | Extant only | 0.35 | 0.31–0.41 |
| Les, Moody & Jacobs (2005) | Australian *Aponogeton* | 17 x 19 | Extant only | 0.36 | 0.30–0.41 |
| Denk & Grimm (2005) | *Zelkova* | 8 x 14 |  | 0.37 | 0.30–0.47 |
| **This study** | **Osmundales** | **122 x 45** | **108** | **0.38** | **0.31–0.43** |
| Denk, Grimm & Hemleben (2005) | *Fagus* | 17 x 42 | 3 | 0.38 | 0.32–0.44 |
| Friis et al. (2009) | Angiosperms | 56 x 114 | 1 | 0.39 | 0.35–0.43 |
| Leht (2009) | *Lathyrus* | 48 x 210 | Extant only | 0.41 | 0.37–0.44 |
| Simpson, Tate & Weeks (2004) | *Hoffmanseggia* | 28 x 33 | Extant only | 0.41 | 0.37–0.45 |
| Hermsen et al. (2006) | Cycadales | 32 x 69 | 18 | 0.47 | 0.36–0.46 |

^a^ This matrix, which has been filtered for apparent homoplasies (Stevenson 1990), is the only matrix that allows inference of a (single) most-parsimonious tree with ample support along branches; the resulting tree, however, is in conflict with molecular trees (e.g. Nagalingum et al. 2011) of the group.

^b^ This combined molecular-morphological matrix is the only one in the list that allows inference of a neighbour net with pronounced tree-like portions compatible with molecular trees obtained for the group.

**References**

Beyra Matos A, Lavin M. 1999. Monograph of *Pictetia* (Leguminosae-Papilionoideae) and review of the Aeschynomeneae. *Systematic Botany Monographs* 56.

Bortiri E, Van den Heuvel B, Potter D. 2006. Phylogenetic analysis of morphology in Prunus reveals extensive homoplasy. *Plant Systematics and Evolution* 259:53-71.

Denk T, Grimm GW. 2005. Phylogeny and biogeography of *Zelkova* (Ulmaceae sensu stricto) as inferred from leaf morphology, ITS sequence data and the fossil record. *Botanical Journal of the Linnéan Society* 147:129-157.

Denk T, Grimm GW, Hemleben V. 2005. Patterns of molecular and morphological differentiation in *Fagus*: implications for phylogeny. *American Journal of Botany* 92:1006-1016.

Friis EM, Crane PR, Pedersen KR, Bengtson S, Donoghue PCJ, Grimm GW, Stampanoni M. 2007. Phase-contrast X-ray microtomography links Cretaceous seeds with Gnetales and Bennettitales. *Nature* 450:549-552.

Friis EM, Pedersen KR, von Balthazar M, Grimm GW, Crane PR. 2009. *Monetianthus mirus* gen. et sp. nov., a nymphaealean flower from the early Cretaceous of Portugal. *International Journal of Plant Sciences* 170:1086-1101.

Gandolfo MA, Nixon KC, Crepet WL. 2004. Cretaceous flowers of Nymphaeaceae and implications for complex insect entrapment pollination mechanisms in early Angiosperms. *Proceedings of the National Academy of Sciences, USA* 101:8056-8060.

Grimm GW. 1999. Phylogenie der Cycadales.Diploma thesis. Eberhard Karls Universität.

Hermsen EJ, Taylor TN, Taylor EL, Stevenson DM. 2006. Cataphylls of the Middle Triassic cycad *Antarcticycas schopfii* and new insights into cycad evolution. *American Journal of Botany* 93:724–738.

Jordal BH, Normark BB, Farrell BD, Kirkendall LR. 2002. Extraordinary haplotype diversity in haplodiploid inbreeders: phylogenetics and evolution of the bark beetle genus *Coccotrypes*. *Molecular Phylogenetics and Evolution* 23:171-188.

Leht M. 2009. Phylogeny of Old World *Lathyrus* L. (Fabaceae) based on morphological data. *Feddes Repertorium* 120:59–74.

Lehtonen S, Myllys L. 2008. Cladistic analysis of *Echinodorus* (Alismataceae): simultaneous analysis of molecular and morphological data. *Cladistics* 24:218-239.

Les DH, Moody ML, Jacobs SWL. 2005. Phylogeny and systematics of *Aponogeton* (Aponogetonaceae): The Australian species. *Systematic Botany* 30:503-519.

Manos PS, Soltis PS, Soltis DE, Manchester SR, Oh S-H, Bell CD, Dilcher DL, Stone DS. 2007. Phylogeny of extant and fossil Juglandaceae inferred from the integration of molecular and morphological data sets. *Systematic Biology* 56:412-430.

Nagalingum NS, Marshall CR, Quental TB, Rai HS, Little DP, Mathews S. 2011. Recent synchronous radiation of a living fossil. *Science* 334:796–799.

Simpson BB, Tate JA, Weeks A. 2004. Phylogeny and character evolution of *Hoffmannseggia* (Caesalpinieae: Caesalpinoideae: Leguminosae). *Systematic Botany* 29:933-946.

Stevenson DM. 1990. Morphology and systematics of the Cycadales. *Memoirs of the New York Botanical Garden* 57:8–55.
